# Effectiveness of Papain-Based Organic Dentifrices Versus Commercial Whitening Dentifrice on Tea-Induced Tooth Stains: An In Vitro Study

**DOI:** 10.7759/cureus.69225

**Published:** 2024-09-11

**Authors:** Sujata Chhabile, Prashanth Vishwakarma, Anoli Agrawal, Shruti R Pundkar, Gaurav Mali, Swapnali Patil, Seema Gupta

**Affiliations:** 1 Department of Public Health Dentistry, Jawahar Medical Foundation's Annasaheb Chudaman Patil Memorial Dental College, Dhule, IND; 2 Department of Public Health Dentistry, Vidarbha Youth Welfare Society's Dental College, Amravati, IND; 3 Department of Orthodontics, Kothiwal Dental College and Research Centre, Moradabad, IND

**Keywords:** bromelain, papain, spectrophotometry, tea, tooth bleaching

## Abstract

Introduction: Tooth discoloration is a common issue for oral health practitioners. Whitening treatments remove surface and deep stains using either chemicals or over-the-counter products. Due to harmful chemical effects, organic whitening products are increasingly preferred. This study compared the effectiveness of organic papain-based dentifrices (Perfora Magic Teeth Whitening Serum, Gurugram, India) on tea stains to commercially available Colgate whitening dentifrices (Colgate-Palmolive, New York, NY).

Methods: Sixty maxillary central incisors for periodontal reasons were used and divided into two groups. Each sample was soaked in freshly brewed tea for 10 minutes daily and stored in artificial saliva (Nanochemazone, Kurukshetra, India) for 24 hours. After four weeks, group 1 was treated with papain-based dentifrices, and group 2 with Colgate Visible White teeth whitening paste (CVWP) daily for four weeks. Spectrophotometric analysis (VITA Easyshade V, VITA Zahnfabrik, Bad Säckingen, Germany) was performed pre-treatment and at four weeks post-treatment. Data were compiled and statistically analyzed.

Results: Compared with group 1, group 2 exhibited an optimal color change (ΔE). Statistically significant differences were observed between the mean ΔL (lightness) and Δb (blueness -b or yellowness +b) values.

Conclusion: CVWP showed better color achievement and stain reduction due to abrasive and peroxide components. Although Perfora Teeth Whitening Serum has low efficacy in upgrading the color of the enamel surface, it is safe for long-term use.

## Introduction

In modern society, having natural teeth and a perfect smile is a prerequisite for visual appeal. People may find it uncomfortable when their natural teeth become discolored. Tooth discoloration is primarily due to intrinsic or extrinsic stains, the underlying causes of which include various health conditions that develop either before or after birth, such as fluorosis or tetracycline stains [1]. Extrinsic stains result from consuming coffee, red wine, turmeric milk, tea, etc. Tea is one of the most popular beverages in the world; it contains tannins, a chemical molecule that is a member of a broad class of substances known as polyphenols. Tannin accumulates on the tooth surface, resulting in extrinsic discoloration of the teeth, which might worsen the condition in the presence of salivary proteins, and tannin adheres to the hydroxyapatite component of enamel [2].

Oral health professionals frequently encounter tooth discoloration. Discoloration can be treated using various in-office procedures, including scaling, abrasive paste polishing, bleaching, and veneer or crown construction [1]. However, these procedures are expensive and need to be performed only by an oral health specialist. A broad variety of in-house teeth whitening products are currently available to meet the requirements of patients. These products have been considered alternatives to professional teeth whitening treatment and may have a lower cost than traditional teeth whitening procedures. These products include Pepsodent dentifrice, Himalayan whitening charcoal, and Colgate Visible White teeth whitening toothpaste (CVWP) (Colgate-Palmolive, New York, NY) [3]. These products primarily include fluorides, thickening agents, solubilizing humectants, solid cleaning abrasive ingredients, and foam-generating surfactants. Preservatives, flavoring agents, buffering and opacifying agents, and hydrogen peroxide are also present. Hydrogen peroxide has proven to be a successful non-invasive treatment for discolored teeth [4]. However, it also has adverse effects, including enamel erosion, increased sensitivity to dental stimulants, gum irritation, and roughening of the tooth surfaces. There is a global need for an alternative treatment option and products for tooth whitening that are free of harmful effects. Natural or chemical-free dentifrices are viable options for reducing extrinsic stains without enamel surface alteration. Tannins adhere to the tooth surface through protein molecules, and cysteine proteases such as papain and bromelain have the ability to hydrolyze protein molecules and have been experimentally proven to reduce surface strain [4,5]. The objective of this study was to compare the effectiveness of a papain-based organic dentifrice (Perfora Magic Teeth Whitening Serum, Gurugram, India) with CVWP in removing tea-induced extrinsic tooth stains in an in vitro setting.

## Materials and methods

Study design

The present in vitro experimental study was conducted at the Department of Public Health Dentistry from January 2024 to July 2024. The study followed the adaptive Minimum Information for Publication of Quantitative Real-Time PCR Experiments (MIQE) [6] and the Checklist for Reporting In Vitro Studies (CRIS) guidelines [7]. This study was approved by the Institutional Ethics Committee of Jawahar Medical Foundation's Annasaheb Chudaman Patil Memorial Dental College (approval number: EC/NEW/INST/2022/2959/2023/048). Written informed consent was obtained from all patients to use the extracted teeth in the experimental study.

The inclusion criteria were sound (caries-free and restoration-free) maxillary central incisor teeth extracted for periodontal reasons. Teeth with caries, developmental defects, enamel discoloration, cracks, fractures, or calcifications were excluded from the study.

The sample size was calculated using G power software version 3.16. Considering a medium effect size of 0.5, 60 samples were obtained at 80% power and an alpha error of 5% [8].

Sample preparation and staining methods

Sixty extracted human maxillary central incisor specimen were equally divided into two groups. Each sample was assigned an individual code using the block randomization method. Debris, calculus, and soft tissue remains were removed from the tooth surfaces using an ultrasonic scaler (Scaler P7, Dentmark, Ludhiana, India). The samples were disinfected with a sodium hypochlorite solution (Denpro Hyposol 3%, Prevest, Jammu, India) for 30 minutes. The teeth were polished with a prophylaxis paste (Denpro Spectra, Prevest, Jammu, India) to eliminate any previously developed extrinsic stains on the tooth surfaces. Subsequently, the samples were kept in a designated group compartment to prevent specimen shuffling.

Tea extract preparation and immersion

**Figure 1:**
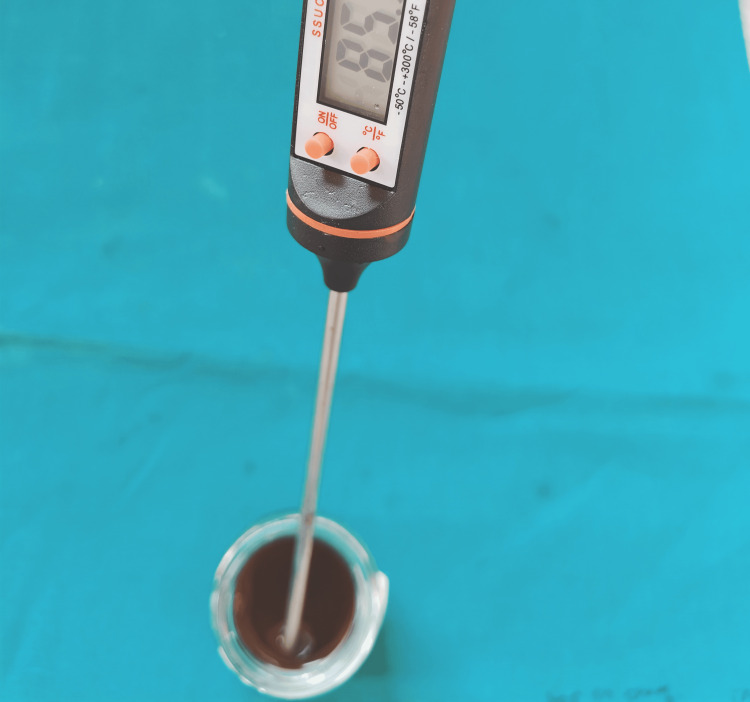
Preparation of tea extract at 85°C

The tea extract solution was prepared by dissolving 9 g of tea powder (Red Label, Hindustan Unilever Limited, Mumbai, India) in 200 mL of boiling water at 100°C. The water temperature was standardized using a portable digital probe thermometer (Romson, Delhi, India) (Figure 1).

The tea extract was cooled to 85°C before sample immersion to mimic the typical temperature of a tea serving [2,9]. The samples were submerged in the tea extract for 10 minutes each day, as the usual tea consumption lasts approximately 10 minutes. They were then preserved in artificial saliva (Nanochemazone, Kurukshetra, India) for 24 hours. This process was performed once a day for the next four weeks. Fresh tea solution was prepared daily prior to tooth immersion.

Brushing protocol

**Figure 2:**
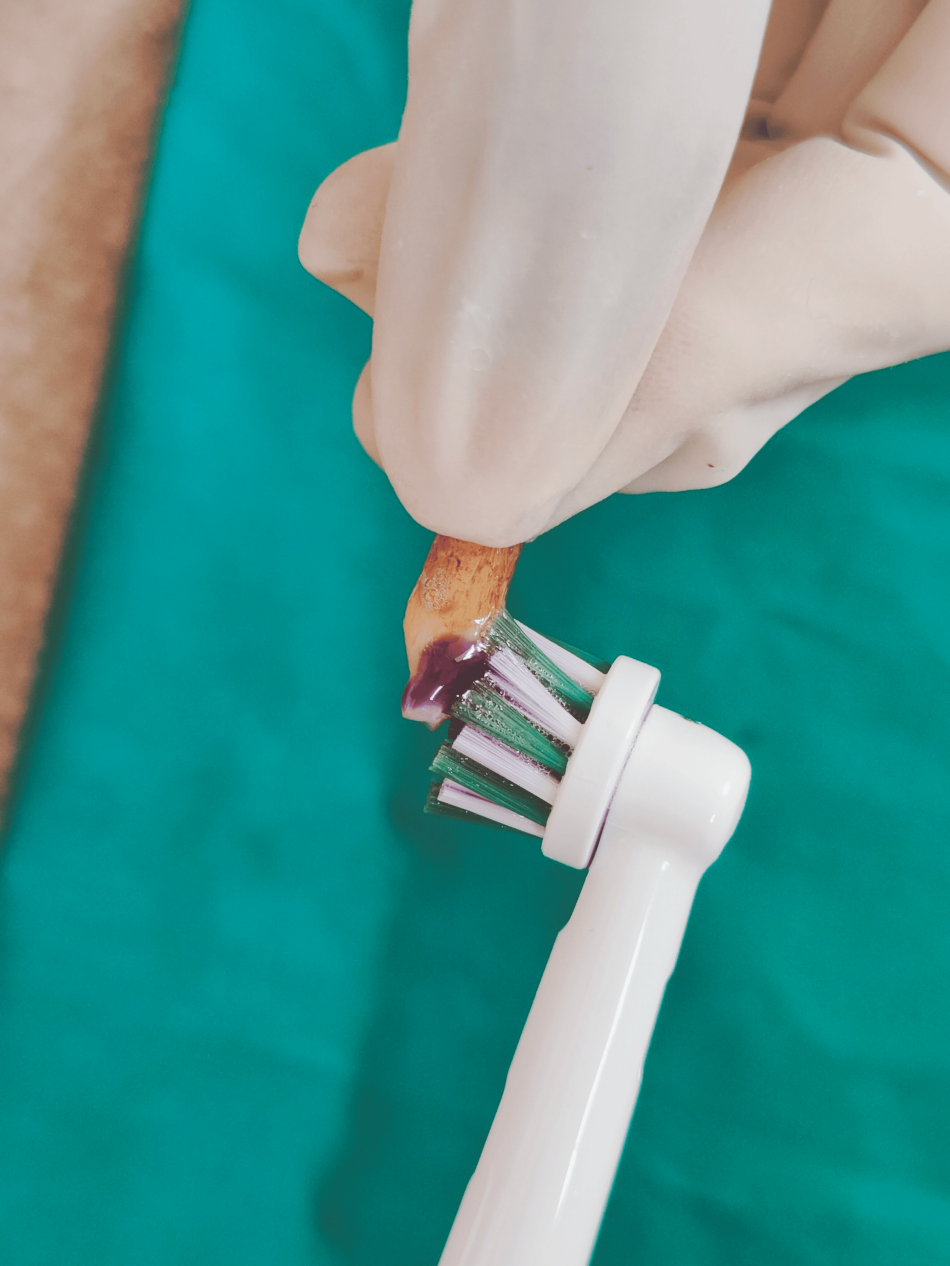
Brushing with electric motorized brush

After four weeks of the staining procedure, the brushing of each sample was scheduled. The recommended brushing duration was two minutes, and the maximum amount of time a single tooth was in contact was 12 seconds [10]. Dentifrice slurries were prepared by combining the allotted dentifrice (Table 1) in a 3:1 ratio with artificial saliva [11]. After applying the slurry to the surface of the crown, the crown was brushed for 12 seconds (six seconds on the buccal and six seconds on the palatal surfaces) and then kept in artificial saliva. To standardize the brushing force, an Oral B® PRO 2 2000 cross-action electric rechargeable toothbrush (Procter & Gamble, Cincinnati, OH) with pressure sensitivity was used (Figure 2).

**Table 1:** Composition of dentifrices to prepare slurry with artificial saliva

Dentifrices	Composition
Colgate Visible White	Silica, sorbitol, glycerin, polyethylene, glycol, sodium tripolyphosphate, tetrapotassium pyrophosphate, sodium lauryl sulfate, flavor, cocamidopropyl betaine, sodium carboxymethyl cellulose, sodium fluoride, xanthan, sodium hydroxide, sorbosil BFG51 blue, titanium dioxide in aqueous base
Perfora Teeth Whitening Serum	Aqua (demineralized water), sorbitol, glycerin, hydrated silica, polysorbate 80, erythritol, sodium carboxymethyl, cellulose, nano-hydroxyapatite, flavor, xylitol, apple fruit extract, peach fruit extract, papain enzyme, bromelain enzyme, sodium methyl cocoyl taurate, cocamidopropyl betaine, potassium sorbate, menthol(crystals) CI 17200, peppermint (essential oil), spearmint (essential oil), stevia, CI 42090

It included a circular brush head with 16-degree angled bristles that operated in three different ways: oscillating, rotating, and pulsing. Brushing strokes were applied as suggested by the manufacturer and were standardized for all specimens. Brushing was also done for four weeks.

Spectrophotometric analysis and data collection

**Figure 3:**
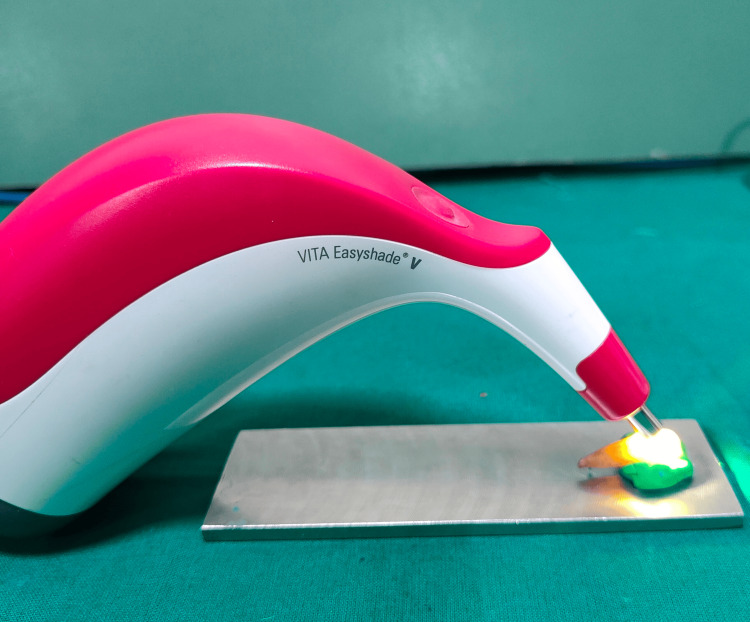
Spectrophotometric analysis of tooth surface

A digital spectrophotometer (VITA Easyshade V, VITA Zahnfabrik, Bad Säckingen, Germany) was used to measure the color on a white background (Figure 3).

The samples were dried using blotting paper prior to color assessment. To hold the teeth in place while they underwent spectrophotometric examination, a thick mold and steel scale were utilized. The central area of the crown on the buccal surface was selected to focus on spectrophotometric readings and standardization for pre- and post-treatment comparisons. The L* a* and b* values (L* indicates lightness, a* is the red/green coordinate, and b* is the yellow/blue coordinate) were measured at baseline (T0) before brushing and after four weeks of brushing (T1). Color changes from tea staining (ΔEpre) to the whitening process (ΔEpost) were calculated using the CIEDE2000 (La Commission Internationale de l’Éclairage) L*a*b* system with the CIE standard illuminant [12]: ΔEpre = {(ΔLpre*)2+ (Δapre*)2 + (Δbpre*)2 }1/2, and ΔEpost = {(ΔLpost*)2 + (Δapost*)2 + (Δbpost*)2 }1/2.

Statistical analysis

The collected data were entered into an MS Excel sheet (Microsoft Corp., Redmond, WA) for statistical analysis using the Statistical Package for the Social Sciences version 23 for Windows (IBM SPSS Statistics, Armonk, NY). Quantitative data were presented as mean and standard deviation (SD). The Shapiro-Wilk test was used to assess the normality of the data distribution. Pre- and post-treatment data were compared using two-way paired t-tests, and intergroup comparisons were made using independent t-tests. The significant value for alpha was set to 0.05.

## Results

The study assessed the changes in color parameters (L* a* b*, and E) before and after treatment in the two groups. In group 1, there was a significant increase in the mean L* value from 67.42 ± 3.06 pre-treatment to 72.72 ± 3.94 post-treatment, with a 95% confidence interval (CI) for the mean difference ranging from -7.77 to -2.82 (p = 0.001, effect size = 1.53). The a* value showed a significant decrease from 4.56 ± 0.90 to 1.71 ± 0.42, with a 95% confidence interval (CI) for the mean difference of 2.28 to 3.42 (p = 0.001, effect size = 3.58). The b* value showed no significant change (p = 0.694, effect size = 0.12). The E value significantly increased from 73.94 ± 3.49 to 78.54 ± 5.70, with a CI for the mean difference between -8.25 and -0.94 (p = 0.019, effect size = 0.89).

In group 2, the L* value significantly increased from 70.02 ± 4.30 pre-treatment to 81.15 ± 4.74 post-treatment, with a CI for the mean difference between -13.45 and -8.81 (p = 0.001, effect size = 3.43). The a* value significantly decreased from 4.68 ± 0.97 to 1.38 ± 0.82, with a CI for the mean difference between 2.57 and 4.02 (p = 0.001, effect size = 3.26). The b* value significantly decreased from 28.94 ± 2.24 to 23.59 ± 4.36, with a CI for the mean difference between 3.08 and 7.61 (p = 0.001, effect size = 1.69). Finally, the E value significantly increased from 75.98 ± 3.41 to 84.66 ± 3.99, with a CI for the mean difference between -10.71 and -6.64 (p = 0.001, effect size = 3.05) (Table 2).

**Table 2:** Comparison of pre- and post-intervention data within the study groups The L* value represents the lightness of the color from 0 to 100, the a* value represents the position of the color between green (-) and red (+), the b* value represents the position of the color between blue (-) and yellow (+), and E represents the Euclidean distance in the color space. †Paired t-test (p < 0.05: significant) Data are presented as mean ± SD. SD: standard deviation

Group	Parameter	Pre-treatment	Post-treatment	95% confidence interval for mean difference		p-value†	Effect size
		Mean ± SD	Mean ± SD	Lower	Upper		
Group 1	L*	67.42 ± 3.06	72.72 ± 3.94	-7.77	-2.82	0.001	1.53
	a*	4.56 ± 0.90	1.71 ± 0.42	2.28	3.42	0.001	3.58
	b*	29.96 ± 2.52	29.18 ± 6.75	-3.56	5.12	0.694	0.12
	E	73.94 ± 3.49	78.54 ± 5.70	-8.25	-0.94	0.019	0.89
Group 2	L*	70.02 ± 4.30	81.15 ± 4.74	-13.45	-8.81	0.001	3.43
	a*	4.68 ± 0.97	1.38 ± 0.82	2.57	4.02	0.001	3.26
	b*	28.94 ± 2.24	23.59 ± 4.36	3.08	7.61	0.001	1.69
	E	75.98 ± 3.41	84.66 ± 3.99	-10.71	-6.64	0.001	3.05

The study compared the color change in parameters (ΔL*, Δa*, Δb*, and ΔE) between two groups. For ΔL*, group 1 had a mean change of 5.3 ± 3.45 with a standard error (SE) of 1.09, a coefficient of variation of 0.65, and a Cohen's d value of -1.74. Group 2 showed a higher mean change of 11.13 ± 3.23 with an SE of 1.02 and a coefficient of variation of 0.29. The difference in ΔL* between the groups was statistically significant (p = 0.001). For Δa*, group 1 exhibited a mean change of -2.85 ± 0.79 with an SE of 0.25 and a coefficient of variation of -0.27, while group 2 had a slightly higher mean change of -3.3 ± 1.01 with an SE of 0.32 and a coefficient of variation of -0.30. The difference in Δa* between the groups was not statistically significant (p = 0.284), with a Cohen's d of 0.49. For Δb*, group 1 had a mean change of -0.78 ± 6.07 with an SE of 1.92 and a high coefficient of variation of -7.79, compared to group 2, which had a mean change of -5.35 ± 3.16, an SE of 1.00, and a coefficient of variation of -0.59. The difference in Δb* was statistically significant (p = 0.049) with a Cohen's d of 0.94. Lastly, for ΔE, group 1 demonstrated a mean change of 4.59 ± 5.11 with an SE of 1.61 and a coefficient of variation of 1.11, whereas group 2 showed a mean change of 8.67 ± 2.83 with an SE of 0.89 and a coefficient of variation of 0.32. The difference in ΔE between the groups was statistically significant (p = 0.041), with a Cohen's d value of -0.98 (Table 3).

**Table 3:** Comparison of change in color between the study groups The ΔL* value represents the difference in post- and pre-treatment lightness of the color, the Δa* value represents the difference in post- and pre-treatment position of the color between green and red, the Δb* value represents the difference in post- and pre-treatment position of the color between blue and yellow, and ΔE represents the change in the Euclidean distance. †Independent t-test (p<0.05: significant) Data are presented as mean ± SD. SD: standard deviation

Parameters	Group	Number	Mean±SD	Standard error	Coefficient of variation	p-value†	Cohen's d
ΔL*	Group 1	30	5.3 ± 3.45	1.09	0.65	0.001	-1.74
	Group 2	30	11.13 ± 3.23	1.02	0.29		
Δa*	Group 1	30	-2.85 ± 0.79	0.25	-0.27	0.284	0.49
	Group 2	30	-3.3 ± 1.01	0.32	-0.30		
Δb*	Group 1	30	-0.78 ± 6.07	1.92	-7.79	0.049	0.94
	Group 2	30	-5.35 ± 3.16	1.00	-0.59		
ΔE	Group 1	30	4.59 ± 5.11	1.61	1.11	0.041	-0.98
	Group 2	30	8.67 ± 2.83	0.89	0.32		

## Discussion

The common causes of extrinsic staining of teeth are red wine, coffee, and tea. Experimentally, tea stains can be easily produced in vivo or in vitro and studied [13]. The present study analyzed the ability of a papain-based dentifrice (Perfora) to improve the color of the enamel surface and compared it with that of the CVWP group. Our results showed that CVWP had better results than papain-based dentifrice.

Both treatments significantly improved lightness (L*) and reduced redness (a*), with CVWP causing a more substantial change. It also reduced yellowness (b*), which did not significantly change in the Perfora group. Both treatments showed a significant overall color change (E value), with CVWP having a larger effect size, indicating a more noticeable difference in tooth color after treatment. CVWP's treatment was more effective at lightening teeth, reducing yellowness, and producing a greater overall color change. This might be due to the fact that the main constituents of CVWP include pyrophosphate silica, sodium lauryl sulfate (SLS), sodium fluoride, sodium hydroxide, and titanium dioxide. The main advantages of titanium dioxide are its high refractive index, brightness, and resistance to discoloration. Hydrated silica is a medium-hard abrasive that aids in stain removal, and sodium lauryl sulfate is a surfactant that inhibits the adhesion of particles to the tooth surface [14]. As CVWP is peroxide-free, silica particles, sodium lauryl sulfate, and cocamidopropyl betaine in applied dentifrices could be responsible for the color change [15]. In contrast, another study comparing papain and bromelain enzymes with Contrast PM+ (Discus Dental, LLC), which is based on 20 wt% carbamide peroxide, showed that carbamide peroxide has a greater effect than papain and bromelain enzymes. Dentifrice containing peroxides releases highly reactive free radicals, leading to the oxidation of organic chromophores and small molecules from coffee, red wine, or tea. They are broken down into smaller molecules and absorb fewer wavelengths of visible light, resulting in a lighter appearance of the teeth [4].

Over-the-counter products are easily available on the market at low cost, but the harmful effects of these products such as gum irritation and tooth sensitivity remain unaltered. However, chemical dentifrices with peroxides are not suitable for long-term use. Studies have shown that the relative dentine abrasiveness of whitening dentifrices varies depending on the composition of the paste and the usage period [16]. The present study showed that the ΔL* (lightness) of the enamel surface achieved by CVWP (mean difference of -11.13) was better than that achieved by Perfora (mean difference of -5.3). CVWP is composed of silica, sodium hydroxide, and titanium oxide, which pertain to the light color of the enamel surface [17].

A clinical study showed that papain and bromelain enzymes are more effective than abrasive whitening of dentifrices. The abrasive action of the toothbrush removes the outer stained plaque but does not change the color of the teeth. Papain is a proteolytic enzyme that breaks down the protein pellicle on the surface of teeth and removes stains that impart color change [3]. Our study used CVWP, which contains silica and sodium pyrophosphate as an ingredient. Silica as abrasive particles have additive effects with sodium pyrophosphate on stain removal, along with color change [18]. In addition to dentifrices, an electric toothbrush was employed in this trial because studies have shown that these brushes are more effective than manual toothbrushes in eliminating stains and maintaining tooth whitening [19].

The present study showed that the Δb* value was significantly lower in Perfora Teeth Whitening Serum than in CVWP, as it contains CI 42090, which gives a blue color, and a blue color on the teeth can counteract the yellow stains on the surface and make teeth appear whiter [3]. The perception of yellow discoloration of teeth is altered by the use of blue covarine and other optically affecting pigments in whitening toothpaste, such as FD&C Blue No. 1, by depositing a thin, semi-transparent blue layer on the dental enamel. Blue is an antagonist of yellow in the color spectrum and causes the net color to shift toward white, resulting in the appearance of whiter, brighter teeth. Within the CIELab color space, the blueshift of the yellow-blue axis (b*) is more significant for the perception of whiter and brighter teeth than changes in the green-red axis (a*) or an increase in brightness (L*) [12].

Although Perfora Teeth Whitening Serum has low efficacy in upgrading the color of the enamel surface, it is safe for long-term use. CVWP contains titanium dioxide and sodium hydroxide (caustic soda), which can increase surface roughness, gum irritation, and sensitivity [4]. Numerous in vivo investigations have demonstrated that TiO2 can injure vital organs by accumulating after entering the bloodstream, and the presence of SLS increases gingival blood flow, indicating that TiO2 can pass through mucous membranes, resulting in oral mucosal epithelial peeling or desquamation, often known as oral epitheliolysis [20,21].

Perfora Teeth Whitening Serum is devoid of titanium dioxide, sodium lauryl sulfate, sodium fluoride, and sodium hydroxide and is safe and certified for use in safe cosmetics in Australia. In contrast to SLS, they use less irritating surfactants such as cocamidopropyl betaine and sodium methyl cocoyl taurate and an alternative to sodium fluoride; they use nano-hydroxyapatite, which is biocompatible and does not induce toxicity, such as the issue of overexposure and ingestion of fluoride, known as fluorosis. Moreover, the gel form of Perfora Teeth Whitening Serum allows it to reach the proximal surface of teeth more easily than abrasive toothpaste [3].

Strengths of the present study

The key strength of the present study was the use of a spectrophotometer for color assessment, which is a more reliable method than the visual method. The study also reports effect sizes (Cohen's d), confidence intervals, and p-values, which strengthen the interpretation of the findings by quantifying the magnitude of the effects and the precision of the estimates. Overall, the study's strengths lie in its robust design, objective measurements, and comprehensive statistical analysis, which together provide reliable, quantifiable results about the effects of the two whitening treatments.

Limitations

The limitation of the study was primarily an in vitro procedure that could not have a realistic oral environment. Only one type of stain was studied in the present research (tea stain). Along with the assessment of change in the color of surface enamel, the susceptibility of the tooth surface to stain after brushing with dentifrice could also be done. Further studies are warranted in this context.

## Conclusions

Papain- and bromelain-based dentifrices hydrolyze the protein pellicle on the tooth surface and reduce the staining of teeth, but the CVWP showed better color achievement and stain reduction due to abrasive and peroxide components. Compared with chemical dentifrices, nonabrasive dentifrices with enzymatic mechanisms are useful for longer spans without harming the tooth surface. The present study showed that the ΔL* (lightness) of the enamel surface achieved by CVWP was better than that achieved by Perfora. However, the Δb* value was significantly lower in Perfora Teeth Whitening Serum than in CVWP.
